# Comparative Analysis of Stress Distribution through Finite-Element Models of 3 NiTi Endodontic Instruments while Operating in Different Canal Types

**DOI:** 10.30476/DENTJODS.2022.90785.1522

**Published:** 2023-03

**Authors:** Maryam Gharechahi, Shaghayegh Moezzi, Sahar Karimpour

**Affiliations:** 1 Dept. of Endodontics, Faculty of Dentistry, Mashhad University of Medical Sciences, Mashhad, Iran; 2 Dentist, Private Practice, Mashhad, Iran

**Keywords:** Endodontics, Finite element analysis, Root canal preparation, Stress analysis

## Abstract

**Statement of the Problem:**

Distribution of stress along endodontic instruments determines their fracture resistance during instrumentation of root canals. The cross-sectional design of instruments and root canal anatomy are of the most important factors affecting the stress distribution.

**Purpose:**

The purpose of this study was to evaluate the stress distribution in different cross-sectional design of nickel-titanium (NiTi) endodontic instruments operating in different canal anatomies using finite element analysis (FEA).

**Materials and Method:**

In this original finite element analysis study, 3-dimensional models of convex triangle (CT), S-type (S), and triple-helix (TH) cross-sectional designs with the size of 25/04 simulated rotational movements through 45ᵒ and 60ᵒ angled root canals with 2- and 5-mm radii using ABAQUS software. The stress distribution was evaluated by the means of FEA.

**Results:**

CT showed the lowest stress values followed by the TH and S ones. The most stress concentration was detected in the CT apical third while, TH revealed better stress distribution all along its length. 45ᵒ curvature angle and 5-mm radius applied the lowest stress to the instruments.

**Conclusion:**

Higher value of radius and smaller curvature angle apply lower stress values to the instrument. CT design shows the lowest stress level with the most stress concentration in its apical third while the triple-helix design has a better stress distribution. Thus, it is safer to use convex triangular cross-section mostly for coronal and middle thirds in initial steps of shaping and triple-helix for the apical third in final steps.

## Introduction

Nickel-titanium (NiTi) rotary instruments have been introduced in 1990s and since then, they have brought root canal instrumentation more effectiveness and speed [ 1 ] and also reduced risk of transportation because of their excellent flexibility [ 2 ]. However, despite many advantages, intracanal separation still occurs with any rotary instrument that can lower the prognosis of the endodontic treatment by leaving the infectious tissue apical to the fractured segment [ 3 ]. Instrument separation is due to two main factors. The first factor is the cyclic fatigue caused by reappearance of bending stresses in curved canals. The second factor is torsion produced within the rotary file when it is blocked against the canal wall, or proposed to disproportionate pressure by the operator [ 1 ]. As the ease of application and effectiveness of rotary instrumentation have increased, its employment by general and other dental practitioners has increased; hence, the intracanal separation has become a matter of concern [ 4 ].

Many studies have been carried out to find the most fracture resistant rotary system and the reasons causing fractures [ 5 - 7 ] and have reported that the design and the manufacturing processes are the main factors determining the mechanical performance of NiTi instruments. There are various rotary systems available in the market with different cross-sectional geometries and still, there is scarce evidence proving which cross-section is more appropriate for which root canal anatomy. Some studies have evaluated cyclic fatigue resistance of endodontic instruments by traditional experimental approaches [ 8 - 11 ]. However, the mechanical behavior of rotary instruments can also be analyzed by finite element analysis (FEA), a numerical method to analyze the stress distribution and concentration in NiTi rotary instruments, which are impossible to be evaluated during actual instrumentation [ 1 ].

The purpose of the present study was to evaluate the stress distribution of three rotary instruments with different cross-section and equivalent size and taper during insertion into root canals with different curvatures and radii. To reach this goal and to simulate the clinical situation, the geometries of endodontic instruments with 3 commonly used cross-sectional designs and root canals with different radii and curvature angles were accurately reproduced, considering mechanical characteristics of NiTi alloy and dentin elastic behavior. In addition, the modeled instruments simulated the rotational movements through modeled root canals at the suggested speed range for clinical use.

The null hypothesis (H_0_) was that neither canal radius and curvature angle, nor instruments’ cross-sectional design have any effect on stress distribution along the endodontic file.

## Materials and Method

**Figure 1:**
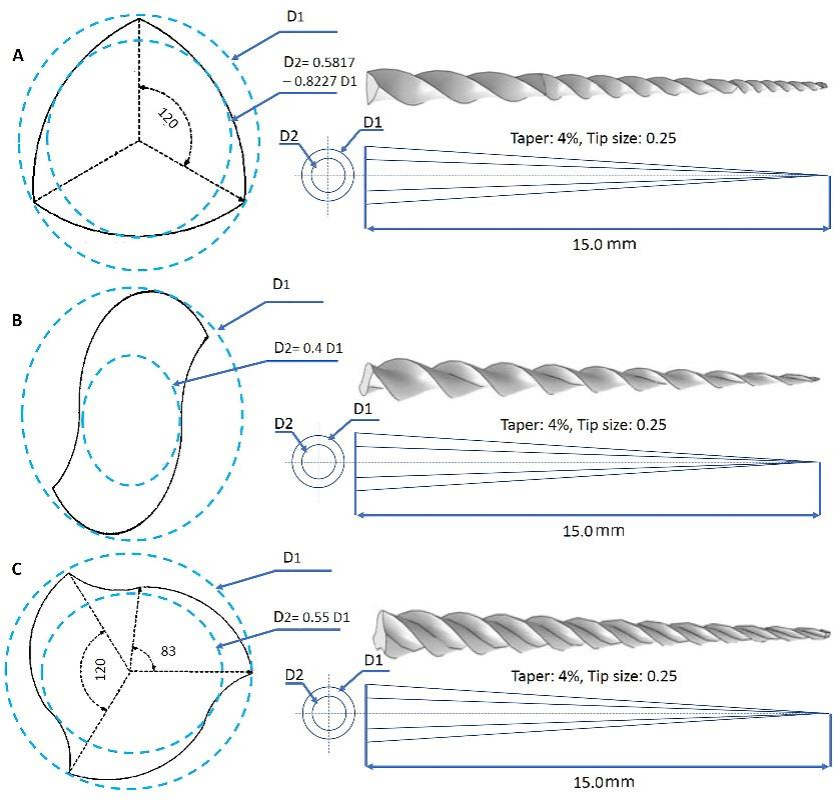
Cross-sectional, longitudinal geometries and finite element models of three NiTi files used in this study. (A) Convex triangle 25/04; (B) S-type 25/04; (C) triple-helix 25/04

Three NiTi rotary systems with different cross-sectional geometries but the same sizes were selected including convex triangle (CT), S-type (S), and triple-helix (TH) with the size and taper of 25/04. Detailed 3-dimentional geometries of these three files were designed using the SolidWorks software (Dassault Systèmes, Vélizy-Villacoublay, France). Cross-sectional and longitudinal geometries of instruments are shown in Figure 1. The zaxis was chosen along the length of the instruments, i.e., normal to the cross section. Root canal models with curvature angles of 45ᵒ and 60ᵒ and radii of 2 and 5mm with the same size as files (25/04) and a total length of 15mm were regenerated according to clinical information.

**Figure 2:**
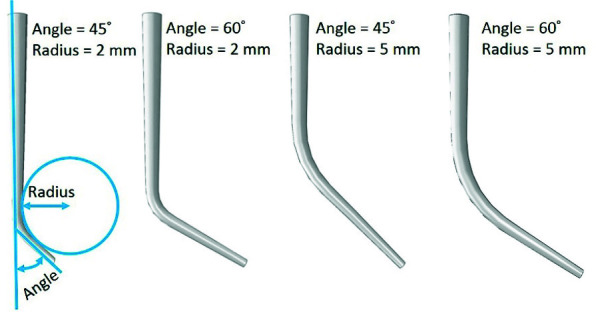
Geometrical models of root canals

By combining the two cited parameters, four types of canal geometries were evaluated (Figure 2). All models were transferred to ABAQUS software V2018 (SIMULIA, Providence, RI, USA) and multiple numerical simulations were performed to evaluate the stress distribution in different endodontic files. Modeled files were advanced continuously, without repetitive up and down pecking or brushing movements to reach the apex of the modeled root canals. During the insertion, the instruments rotated at the speed of 300 rpm (5 revolutions per second) and the von Mises stress distribution was evaluated based on the finite element method.

The mechanical characteristics of NiTi alloy and dentin component of the root canal were setting as: Young’s modulus 36 GPa, the Passion’s ratio 0.30, the stress range for austenite to martensite phase transformation 504-600 MPa for the NiTi alloy [ 12 ] and the Young’s modulus 18.60 and the Poisson’s ratio 0.30 for dentin [ 2 ].

The accumulation of plastic deformation due to cyclic loading in the pseudo-elastic range and the shear strains due to friction of the instrument blade into the canal wall were neglected as model simplifications.

## Results

**Figure 3:**
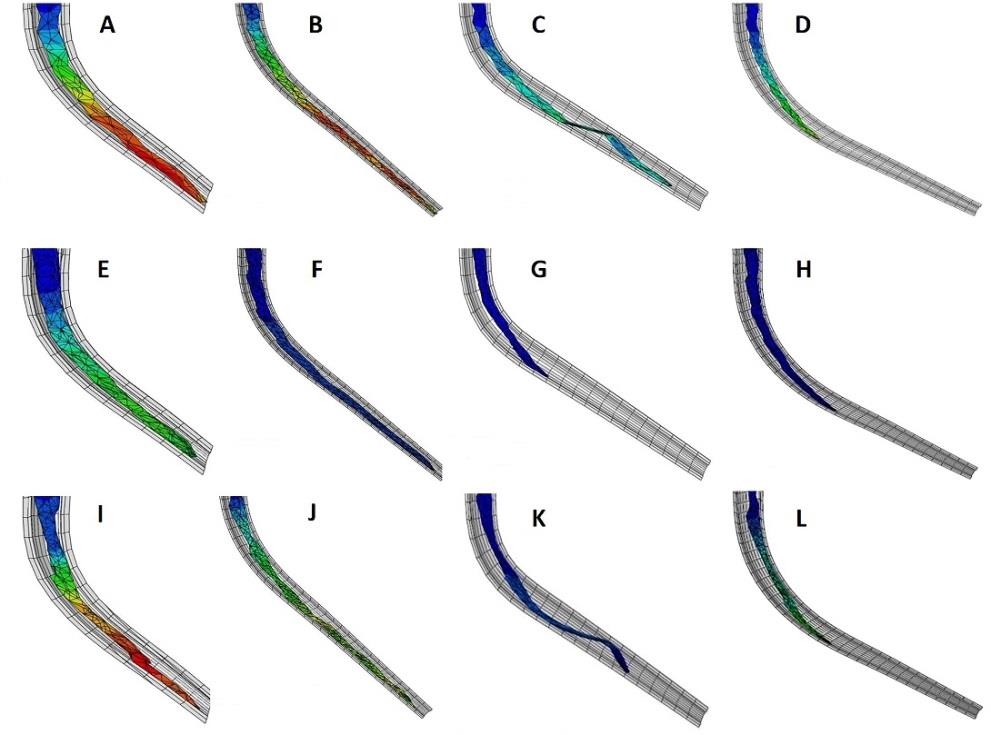
(A-D) convex triangle in 45ᵒ/2mm, 45ᵒ/5mm, 60ᵒ/2mm, and 60ᵒ/5mm root canal models; (E-H) triple-helix in 45ᵒ/2mm, 45ᵒ/5mm, 60ᵒ/2mm, and 60ᵒ/5mm root canal models; (I-L) S-type in 45ᵒ/2mm, 45ᵒ/5mm, 60ᵒ/2mm, and 60ᵒ/5mm root canal models. Lower stress values are shown in blue and higher levels of stress are displayed in red

Figure 3 shows the stress distribution in three instruments while rotating in simulated canals in a longitudinal inner view and Table 1 shows the maximum stress (S_max_) and the minimum stress (S_min_) values of instruments in different canals. The von Mises stress values increased with decreasing radius and increasing curvature angle. The most demanding working condition was related to 2-mm radius and 60ᵒ curvature angle for all 3 instruments while the best operating condition was with 5-mm radius and 45ᵒ curvature angle. CT design passed the 45◦ angled curve without plastic deformity. It showed the lowest stress at the beginning of the curve increased gradually toward the end. The highest stress was recorded in the apical third (475 MPa for 2 mm radius and 397 MPa for 5 mm radius). But in the 60 angled curvature, plastic deformity occurred and the file didn’t pass the curve before fracture.

**Table 1:** Maximum stress (S_max_) and minimum stress (S_min_) values (MPa) recorded for different files in different root canals

Cross section	45ᵒ/2mm		45ᵒ/5mm		60ᵒ/2mm		60ᵒ/5mm	
	S_max_ (MPa)	S_min_ (MPa)	S_max_ (MPa)	S_min_ (MPa)	S_max_ (MPa)	S_min_ (MPa)	S_max_ (MPa)	S_min_ (MPa)
Convex triangle	475	53.2	397	34.3	1620	517	636	219
Triple-helix	610	37.2	400	61.5	1783	275	757	141
S-type	628	90.9	458	166	4180	235	882	278

TH design showed moderate stress in canals with 45ᵒ curvature angle (610 MPa for 2 mm and 400 MPa for 5 mm radius). However, significant deformity occurred in 60ᵒ angle and it did not pass the curves.

The amount of stress recorded for S cross-sectioned instrument was the most in 45ᵒ angle (628 MPa for 2 mm and 458 MPa for 5 mm radius). It did not pass the 60ᵒ-angled curves, too.

## Discussion

This study evaluated the stress distribution and stress concentration in three different rotary instruments. Examination of fractures at high magnification reveals the crack origin and the mode of material failure [ 13 ]. However, it cannot estimate the stress distribution on the instrument that might contribute to its breakage. It is also impossible to evaluate the stresses distributed along the instrument during its actual clinical use. Therefore, a computerized simulation can be useful. In medical and dental research, FEA has been widely used to analyze the stress distribution in complex structures for years [ 14 ]. So, as many other studies [ 1 - 2 , 12 , 15 ], we evaluated the stress distribution along three different endodontic instruments by the means of FEA. Several studies have reported the cross-section profile as the main factor for NiTi instruments torsional behavior [ 5 - 7 ]. In the present study, we regenerated computerized models with different geometries but the same size and taper in different boundary conditions (simulated curved canals) concerning the material mechanical properties. There is no information about “fit” of the instrument in the simulated canals in most articles and some have described the canal diameter wider than the file [ 5 , 16 ]. As the instrument is likely to be fitting loosely in canals, the description of the radius in these studies may be overstated and the file was bent less severely than reported. In addition, most previous studies have not considered the canal taper [ 16 - 18 ]. However, in the present study, we regenerated root canals with the same size and taper as modeled instruments (25/04). Moreover, we chose different cross sections (convex triangle, S-type, and triple-helix) as three common geometries used in endodontic instruments design. Besides, unlike many previous works in the literature, which considered the canal as a rigid body, here the elastic behavior of the canal is taken into account.

Overall, within the limitations of this study and in rejection of the null hypothesis, the increase in the curvature angle and the decrease in canal radius resulted in higher stress values along the instruments. This was in accordance with other studies considering the effect of canal radius on instrument behavior [ 9 , 16 , 19 ]. Radius parameter represents the severeness of the canal curvature. Smaller radius means a more abrupt curve that applies higher stress values to the instrument [ 9 ].

Our study showed that CT design undergoes the lowest stress values through the curves, while S shows the highest stress values and TH showed moderate stress values. It is in accordance with the studies of Berutti *et al*. [ 17 ] and Xu *et al*. [ 7 ] that reported the lower stress values for convex triangle cross-sectioned instrument compared to other instrument designs. In addition, Kim *et al*. [ 20 ] evaluated the mechanical behavior of different cross-sectional designs and reported lower stress concentration in HeroShaper with triple-helix cross-section than Mtwo with an S-type cross-section. These results can be due to the larger inner core in CT design that allows the stress to be spread in a larger area while deeper flutes in TH and S designs results in smaller inner core diameter. Zhang *et al*. [ 18 ] reported similar stress resistance for CT and TH designs but stress value was still higher in S-type cross section. This can be due to different methodologies as they studied the instrument mechanical behavior by applying torsional and bending loads to the instrument tip instead of simulating canals with specific radius and curvature angle.

The highest stress levels were observed in apical third of instruments especially in CT. However, the TH design showed a better stress distribution. In accordance with our results, Basheer Ahmed *et al*. [ 2 ] observed maximum bending stress in the apical third of the convex triangle cross-section. Moreover, Inan *et al*. [ 9 ] reported fracture at the apical third of F2 ProTaper file that has the same cross-sectional profile and the same size as our CT design. The reason can be the increased diameter of CT design. Although CT cross section has a larger inner core to spread the stress but larger inner core results in less flexibility that requires higher loads to bend. This can increase the stress level in the apical section, which is more bent. Accumulation of maximum stress in the apical third of the CT design increases the risk of its fracture and it is safer to use it for coronal and middle thirds shaping.

## Conclusion

Higher value of radius and smaller curvature angle apply lower stress values to the instrument. In this study, 5-mm of radius and 45ᵒ curvature angle was the best operating condition while 2-mm radius and 60ᵒ angle was the worst. Convex triangle design showed the lowest stress level compared with triple-helix and S-type cross sections. Convex triangle design showed the most stress concentration in its apical third while the triple-helix design had a better stress distribution. Thus, it is safer to use convex triangular cross-section mostly for coronal and middle thirds in initial steps of shaping and triple-helix for the apical third in final steps.

## Acknowledgements

This article is based on an undergraduate thesis (No. 2593). The authors would like to thank the Vice Chancellor for Research at Mashhad University of Medical Sciences for technical and financial support.

## Authors’ contributions

The authors certify that all authors have contributed significantly and all are in agreement with the manuscript.

## Conflict of Interest

The authors declare that they have no conflict of interest.
